# BubR1 Insufficiency Impairs Liver Regeneration in Aged Mice after Hepatectomy through Intercalated Disc Abnormality

**DOI:** 10.1038/srep32399

**Published:** 2016-08-26

**Authors:** Ayae Ikawa-Yoshida, Takuya Matsumoto, Shinji Okano, Yukihiko Aoyagi, Yutaka Matsubara, Tadashi Furuyama, Yoshimichi Nakatsu, Teruhisa Tsuzuki, Mitsuho Onimaru, Tomoko Ohkusa, Masatoshi Nomura, Yoshihiko Maehara

**Affiliations:** 1 Department of Surgery and Science, Graduate School of Medical Sciences, Kyushu University, Fukuoka, Japan; 2 Department of Medical Biophysics and Radiation Biology, Graduate School of Medical Sciences, Kyushu University, Fukuoka, Japan; 3 Division of Pathophysiological and Experimental Pathology, Department of Pathology, Graduate School of Medical Sciences, Kyushu University, Fukuoka, Japan; 4 Center for Clinical and Translational Research, Kyushu University, Fukuoka, Japan; 5 Department of Endocrine and Metabolic Diseases / Diabetes Mellitus Kyushu University, Fukuoka, Japan

## Abstract

A delay in liver regeneration after partial hepatectomy (PHx) leads to acute liver injury, and such delays are frequently observed in aged patients. BubR1 (budding uninhibited by benzimidazole-related 1) controls chromosome mitotic segregation through the spindle assembly checkpoint, and BubR1 down-regulation promotes aging-associated phenotypes. In this study we investigated the effects of BubR1 insufficiency on liver regeneration in mice. Low*-BubR1*-expressing mutant (*BubR1*^*L/L*^) mice had a delayed recovery of the liver weight-to-body weight ratio and increased liver deviation enzyme levels after PHx. Microscopic observation of *BubR1*^*L/L*^ mouse liver showed an increased number of necrotic hepatocytes and intercalated disc anomalies, resulting in widened inter-hepatocyte and perisinusoidal spaces, smaller hepatocytes and early-stage microvilli atrophy. Up-regulation of desmocollin-1 (DSC1) was observed in wild-type, but not *BubR1*^*L/L*^, mice after PHx. In addition, knockdown of BubR1 expression caused down-regulation of DSC1 in a human keratinocyte cell line. BubR1 insufficiency results in the impaired liver regeneration through weakened microstructural adaptation against PHx, enhanced transient liver failure and delayed hepatocyte proliferation. Thus, our data suggest that a reduction in BubR1 levels causes failure of liver regeneration through the DSC1 abnormality.

BubR1 (budding uninhibited by benzimidazole-related 1) plays an important role in the spindle assembly checkpoint to prevent chromosome missegregation and aneuploidy during mitosis. BubR1 controls the E3 ubiquitin ligase, termed anaphase-promoting complex/cyclosome (APC/C), until all kinetochores are attached with microtubules in an appropriate manner^1^. When all the kinetochores establish bipolar attachment, APC/C degrades securin and cyclin B, and promotes metaphase-anaphase transition^2^. BubR1 dysfunction causes an unequal segregation of chromosomes, resulting in chromosomal instability. Baker *et al*. found that decreased BubR1 expression induces cellular senescence through p16^INK4a^ up-regulation, and that mutant mice with decreased BubR1 expression display various progeroid phenotypes, such as short lifespan, cachectic dwarfism, lordokyphosis, cataracts, loss of subcutaneous fat and impaired wound healing^3^,^4^,^5^,^6^. Therefore, BubR1 could play an important role in regulating aging. We recently generated a new *BubR1* low-expression mouse, which is a useful model to investigate the role of BubR1 in response to various kinds of stress because they do not display any severe phenotypes during growth and development under normal circumstances^7^. Using this animal model, we have shown that reduced BubR1 expression inhibits intimal hyperplasia mediated by reduced vascular smooth muscle cell proliferation after carotid artery ligation^7^.

Adult hepatocytes are normally quiescent cells in the G0 phase of the cell cycle and do not undergo cell division, whereas they proliferate to maintain liver homeostasis in response to several stimuli, such as surgical resections or liver injury^8^,^9^. After partial hepatectomy (PHx), most of the quiescent hepatocytes (95% in young and 75% in old rats) rapidly enter the cell cycle^8^. In the mouse liver, peak DNA synthesis occurs at about 36–44 h after PHx, and DNA synthesis is synchronously initiated in hepatocytes^8^,^10^,^11^. When DNA synthesis is impaired, hepatic regeneration is also impaired^12^,^13^. Most of the increase in liver mass occurs within 3 days after PHx and the remnant liver regenerates to a size equivalent to the original volume within 5–7 days^14^.

In animal models, hepatocytes are directly damaged and thereby induced to undergo necrosis or apoptosis to eliminate damaged cells after PHx^15^. Hepatocyte proliferation is initiated by several growth factors or cytokines during liver regeneration that occurs after massive hepatocyte necrosis or apoptosis^16^. The liver architecture during regeneration after PHx is significantly changed, and this change impacts liver function. Intra- and inter-cellular junctions temporarily change during regeneration following PHx and reformation of the normal liver architecture occurs only after the original volume is restored. The mechanisms that regulate the reorganization of the liver architecture are not well understood^10^.

Liver regeneration is a series of physio-pathological phenomena that allow recovery of damaged tissue and prevent liver failure^17^. Impairment of liver regeneration is a critical problem for aged patients with liver diseases after surgical resection and PHx because their liver does not have the ability to regenerate physically and functionally. In the clinical setting, impairment of liver regeneration leads to liver dysfunction, which can worsen or affect the patient’s general condition and their postoperative prognosis. The operative mortality rate for patients after major hepatectomy increased incrementally with age^18^. Aging impairs liver regeneration and there is a reduced rate of hepatocyte proliferation following resection^19^. However, the mechanism of impaired regenerative capacity in the aged liver has not been fully elucidated.

A previous study suggests that BubR1 insufficiency causes early onset of aging-associated phenotypes^3^, but the physiological relevance of BubR1 to liver regeneration and/or the effects of BubR1 on liver architecture remain unclear. The purpose of this study is to investigate the effects of BubR1 insufficiency on liver regeneration and its architecture using *BubR1* low-expression mice.

## Results

### BubR1 mRNA expression in liver regeneration after partial hepatectomy

**Figure 1:**
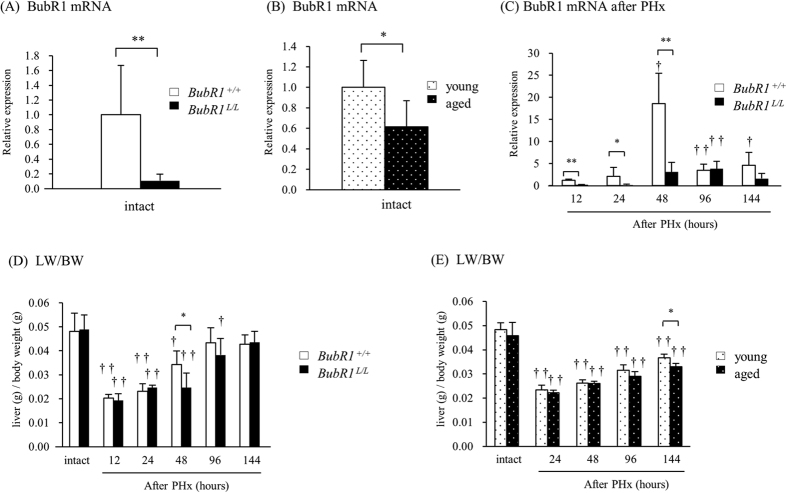
Alterations in BubR1 mRNA expression and liver weight (LW)/body weight (BW) ratio. (**A**) BubR1 mRNA expression in intact *BubR1*^+/+^ (□) and *BubR1*^*L/L*^ (■) mice. (**B**) BubR1 mRNA expression in intact 9-week-old (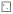) and 55-week-old (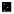) C57BL/6JJcl mice. (**C**) Changes in BubR1 mRNA expression in *BubR1*^+/+^ (□) and *BubR1*^*L/L*^ (■) mice after PHx. Expression levels relative to unhepatectomized *BubR1*^+/+^ mice are shown. (**D**) Changes in LW/BW in *BubR1*^+/+^ (□) and *BubR1*^*L/L*^ (■) mice after PHx. (**E**) Changes in LW/BW in 9-week-old (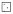) and 55-week-old (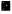) C57BL/6JJcl mice. Data are presented as the mean ± S.D. ^†^p < 0.05, ^††^p < 0.01 vs. intact mouse in each group. *p < 0.05, **p < 0.01 vs. *BubR1*^+/+^ mice at each sampling point.

BubR1 mRNA expression levels in the liver are shown in Fig. 1A,B,C. In *BubR1*^*L/L*^ mice, BubR1 mRNA expression was significantly lower (0.11 ± 0.09, *P* < 0.01) than that of *BubR1*^+/+^ mice (1.00 ± 0.67; Fig. 1A). BubR1 expression (0.62 ± 0.26, *P* < 0.05) was significantly decreased in aged mice compared with young mice (1.00 ± 0.26; Fig. 1B). Figure 1C revealed an alteration of BubR1 mRNA expression after PHx in *BubR1*^*L/L*^ mice and *BubR1*^+/+^ mice. In *BubR1*^+/+^ mice, BubR1 mRNA expression was significantly increased after PHx and reached a maximum level 48 h after PHx (18.55 ± 6.92). In *BubR1*^*L/L*^ mice, the expression level was low, similar to that observed in untreated *BubR1*^*L/L*^ mice. In addition, BubR1 expression was delayed in *BubR1*^*L/L*^ mice.

The LW/BW was compared in *BubR1*^+/+^ and *BubR1*^*L/L*^ mice (Fig. 1D), and in young and aged mice (Fig. 1E). In all groups, LW/BW was significantly decreased 12 h after PHx. LW/BW was significantly lower in *BubR1*^*L/L*^ mice (0.025 ± 0.006, *P* < 0.05) compared with *BubR1*^+/+^ mice (0.034 ± 0.006) 48 h after PHx (Fig. 1D), and significantly lower in aged mice (0.033 ± 0.002, *P* < 0.05) compared with young mice (0.037 ± 0.001) 144 h after PHx (Fig. 1E). The delay in liver regeneration did not cause significant change in mortality between *BubR1*^+/+^ and *BubR1*^*L/L*^ mice at any time point after PHx.

### Biochemical analysis during liver regeneration

Tables 1 and 2 show serial changes in laboratory data of mice with PHx. Plasma AST, ALT and LDH levels were dramatically increased 12 h after PHx in *BubR1*^*L/L*^ and *BubR1*^+/+^ mice, and these values were significantly higher in *BubR1*^*L/L*^ mice than in *BubR1*^+/+^ mice 12‒24 h after PHx (Table 1). In addition, plasma T-BIL, D-BIL, I-BIL, TBA and ALP levels were significantly higher in *BubR1*^*L/L*^ mice 24 h after PHx. These data indicate that, in *BubR1*^*L/L*^mice, impaired liver function continues for a longer period compared with *BubR1*^+/+^ mice. The effect of aging on liver dysfunction after PHx was also examined, and levels of plasma AST, ALT and LDH 24‒48 h after PHx were significantly greater in aged mice compared with those in young mice (Table 2).

### BubR1 insufficiency caused an abnormal cell-cycle progression in hepatocyte after partial hepatectomy

**Figure 2:**
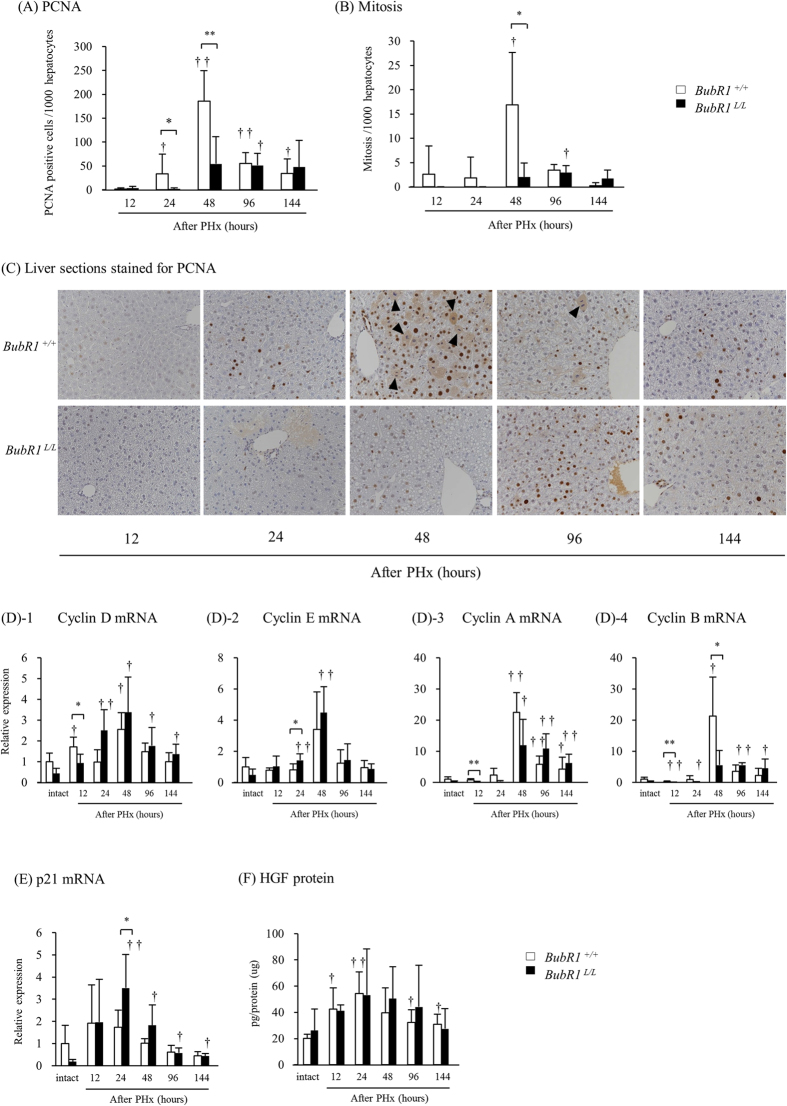
Changes in proliferation markers after PHx. (**A**) PCNA-positive cells, (**B**) cells undergoing mitosis. (**C**) Representative liver sections at 12, 24, 48, 96, and 144 h after PHx stained for PCNA (magnification ×200). Arrowheads, cells undergoing mitosis. (**D**) cyclin D, cyclin E, cyclin A and cyclin B mRNA expression, (**E**) p21 mRNA expression, (**F**) HGF level in *BubR1*^+/+^ (□); *BubR1*^*L/L*^ (■); data are presented as the mean ± S.D. ^†^p < 0.05, ^††^p < 0.01 vs. intact mouse in each group. *p < 0.05, **p < 0.01 vs. *BubR1*^+/+^ mice at each sampling point.

To investigate the underlying mechanisms of impaired liver regeneration in *BubR1*^*L/L*^ mice, we examined alterations of proliferation markers after PHx (Fig. 2A,B). In *BubR1*^+/+^ mice, the number of PCNA-positive hepatocytes (185.7 ± 63.5 vs. 1.69 ± 2.16, *P* < 0.01) and those undergoing mitosis (16.9 ± 10.7 vs. 2.59 ± 5.80, *P* < 0.05) significantly increased 48 h after PHx compared with those 12 h after PHx. Such increases of both values were not observed in *BubR1*^*L/L*^ mice (PCNA, 54.3 ± 56.7; mitosis, 2.0 ± 2.9). Figure 2C shows representative PCNA-stained liver sections from *BubR1*^*L/L*^ and *BubR1*^+/+^ mice at 12, 24, 48, 96, and 144 h after PHx. The peak of hepatocyte proliferation is observed 48 h after PHx in normal mice^20^. Slow progression of hepatocyte proliferation was evident in *BubR1*^*L/L*^ mice 48 h after PHx and thereafter.

Based on reduced hepatocyte proliferation, we also investigated cyclin-D, cyclin-E, cyclin-A and cyclin-B mRNA expression levels (Fig. 2D). In *BubR1*^*L/L*^ mice, cyclin-D (0.93 ± 0.44, *P* < 0.05), cyclin-A (0.23 ± 0.07, *P* < 0.01) and cyclin-B (0.10 ± 0.05, *P* < 0.01) mRNA expression levels were significantly lower compared with those of *BubR1*^+/+^ mice (1.71 ± 0.47, 0.87 ± 0.27 and 0.32 ± 0.13, respectively) 12 h after PHx. The expression patterns of cyclin-D in *BubR1*^+/+^ and *BubR1*^*L/L*^ mice were different: two peaks at 12 and 48 h after PHx were observed in *BubR1*^+/+^ mice while a single but broader peak was observed in *BubR1*^*L/L*^ mice. In *BubR1*^+/+^ mice, cyclin-A and cyclin-B mRNA expression showed remarkable increases 48 h after PHx, and rapidly decreases 96 h after PHx. Cyclin-B mRNA expression was significantly attenuated in *BubR1*^*L/L*^ mice (5.6 ± 4.6, *P* < 0.05) compared with *BubR1*^+/+^ mice (21.3 ± 12.5) 48 h after PHx. These low expression levels continued up to 144 h after PHx without a rapid decrease. Similar patterns were observed in the expression of cyclin-A. Cyclin-E mRNA expression was significantly higher in *BubR1*^*L/L*^mice (1.4 ± 0.4, *P* < 0.05) compared with *BubR1*^+/+^ mice (0.8 ± 0.4) 24 h after PHx.

We also examined the expression of a CDK inhibitor, p21 (Fig. 2E). In *BubR1*^*L/L*^ mice, p21 mRNA expression (3.51 ± 1.51, *P* < 0.05) was significantly higher than that in *BubR1*^+/+^ mice (1.74 ± 0.76) 24 h after PHx. We also examined the mRNA expression of p16^INK4a^, another CDK inhibitor, because it is reported that down-regulation of BubR1 causes up-regulation of p16 ^INK4a^ expression and induces cellular senescence^4^,^5^. In our study, however, p16 ^INK4a^ mRNA expression was too low to detect in mice (data not shown). Moreover, we analyzed levels of HGF that is known to play an important role in driving liver regeneration^21^. There was no significant difference in the level of liver HGF between *BubR1*^+/+^ and *BubR1*^*L/L*^ mice (Fig. 2F).

### BubR1 insufficiency increases hepatocyte necrosis accompanied with intercalated disc abnormality

**Figure 3:**
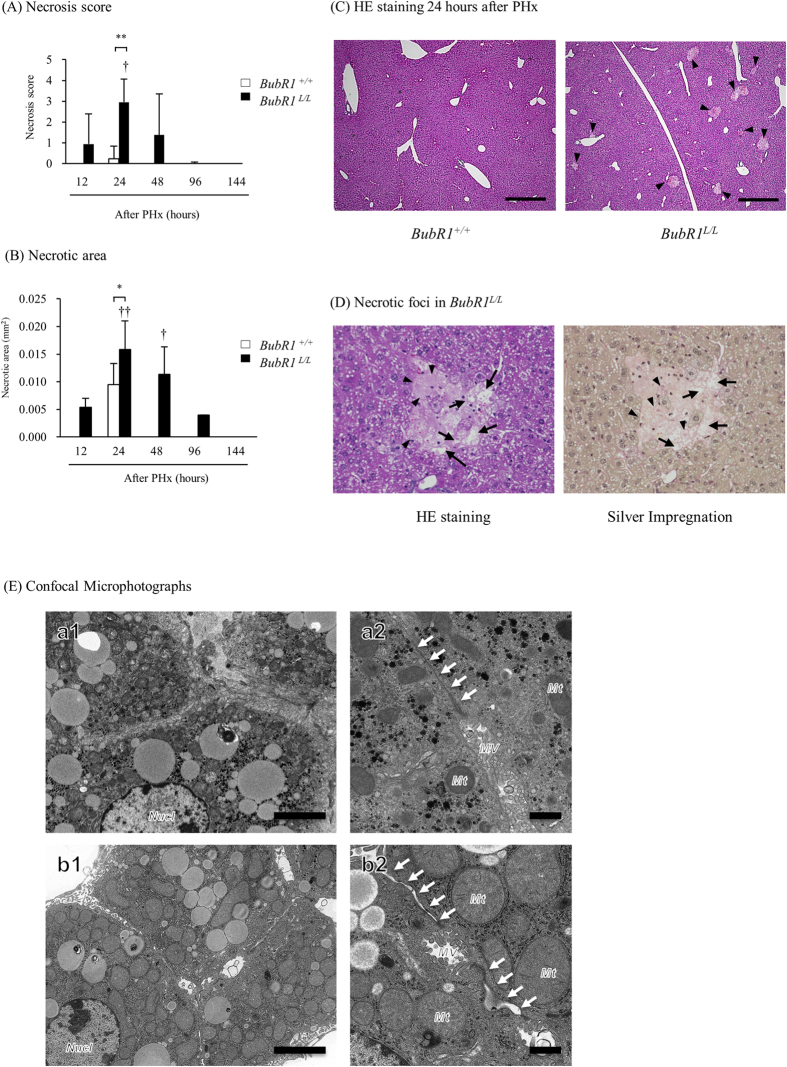
Histopathological data. (**A**) Necrosis score and (**B**) necrotic area after PHx: *BubR1*^+/+^ (□); *BubR1*^*L/L*^ (■); data are expressed as the mean  ±  SD. ^†^p < 0.05, ^††^p < 0.01 vs. 12 h after PHx. *p < 0.05 vs. *BubR1*^+/+^ mice at each sampling point. (**C**) Representative altered liver histopathology at 24 h after PHx with HE staining (magnification ×40): arrowhead, necrosis foci. Scale bars: 500 μm (**D**) Representative necrosis foci histopathology at 24 h after PHx in *BubR1*^*L/L*^ mice: Left, HE staining; right, silver impregnation (magnification ×400); arrow, necrotic hepatocytes; arrowhead, dilation of perisinusoidal spaces with hemorrhages. (**E**) Transmission electron microphotographs of hepatic tissue obtained from *BubR1*^+/+^ (panels a1 and a2) and *BubR1*^*L/L*^ (panels b1 and b2) mice 12 h after PHx. Compared with *BubR1*^+/+^ mice, *BubR1*^*L/L*^ mice displayed a wider intercellular space. Observation at higher magnification revealed a wider perisinusoidal space containing atrophic microvilli and dispatched intercalated discs (white arrows) in the hepatic tissue of *BubR1*^*L/L*^ mice (panel b2). In addition, hepatocyte mitochondria were severely swollen and electron-lucent. Nucl, nucleus; MV, microvilli; Mt, mitochondria; white arrows, intercalated disc. Scale bars: 5 μm in panels a1 and b1; 1 μm in panels a2 and b2.

There were no detectable differences in liver histology between intact *BubR1*^+/+^ and *BubR1*^*L/L*^ mice (data not shown). In *BubR1*^*L/L*^ mice, focal hepatic necrosis was observed 12‒96 h after PHx and the number of necrotic foci in the liver tissue of *BubR1*^*L/L*^ mice (2.95 ± 1.11, *P* < 0.01) was significantly higher than in the *BubR1*^+/+^ mice (0.23 ± 0.61; Fig. 3A) at 24 h after PHx. Also the necrotic area in *BubR1*^*L/L*^ mice (0.016 ± 0.005, *P* < 0.05) was significantly larger than that in *BubR1*^+/+^ mice (0.010 ± 0.004; Fig. 3B) at 24 h after PHx. The necrotic area in *BubR1*^*L/L*^ mice was greatest at 24 h and then gradually decreased. The necrotic foci scattered in liver lobule and it was characterized by necrotic hepatocytes and dilation of perisinusoidal spaces with hemorrhage (Fig. 3C,D).

Figure 3E shows transmission electron microphotographs of hepatic tissue obtained from *BubR1*^+/+^ (panels a1 and a2) and *BubR1*^*L/L*^ mice (panels b1 and b2) 12 h after PHx. Compared with *BubR1*^+/+^ mice, *BubR1*^*L/L*^ mice showed wider intercellular spaces. Observation at higher magnification revealed a wider perisinusoidal space containing atrophic microvilli and dispatched IDs (white arrows) in the hepatic tissue of *BubR1*^*L/L*^ mice (panel b2). In addition, hepatocyte mitochondria were severely swollen and electron-lucent. In *BubR1*^*L/L*^ mice 12 h after PHx, the number of damaged hepatocytes was higher than in *BubR1*^+/+^ mice, and these features were not observed in *BubR1*^*L/L*^ mice without PHx (data not shown). These histopathological findings suggest that in *BubR1*^*L/L*^ mice, abnormal structural intercellular alterations are accompanied by hepatocyte necrosis after PHx.

### Effects of low BubR1 expression on desmocollin-1 expression

**Figure 4:**
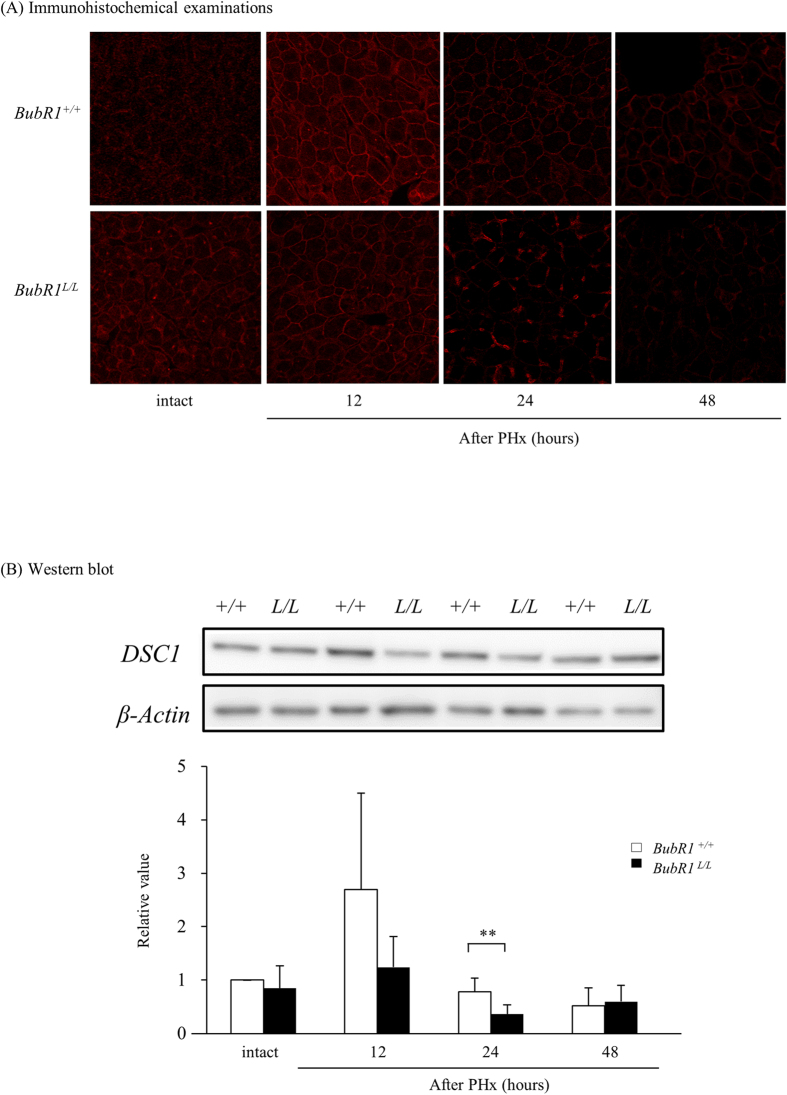
DSC1 expression in hepatocytes after PHx. (**A**) Immunohistochemical staining of DSC1: red staining indicates DSC1 at the intercalated disc. (**B**) Western blotting data of DSC1: upper panel shows representative data for DSC1; □, *BubR1*^+/+^; ■, *BubR1*^*L/L*^; data are mean ± S.D.

**Figure 5:**
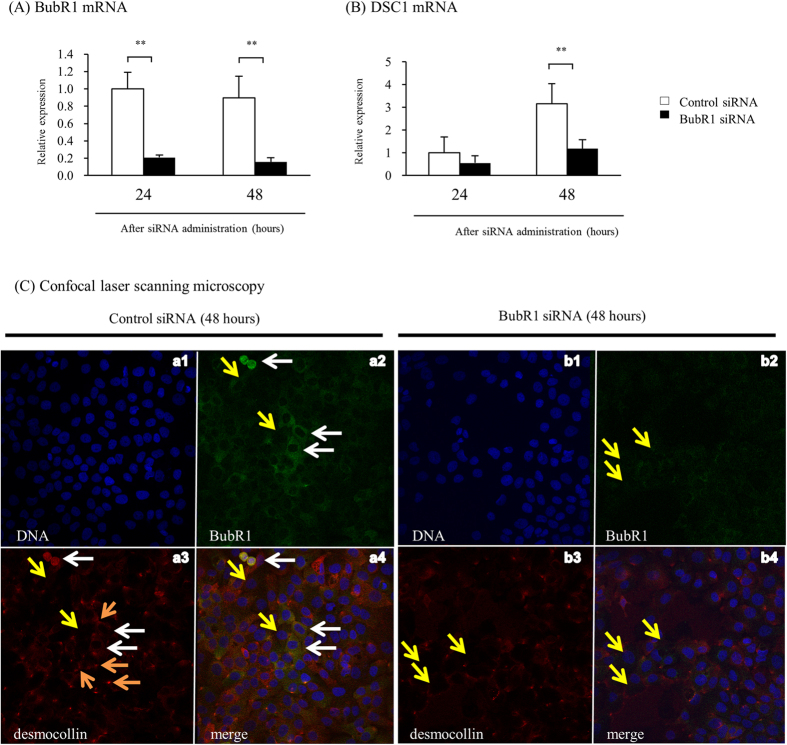
Effect of BubR1 siRNA on DSC1 expression in HaCaT cells. (**A**) Expression of BubR1 and (**B**) DSC1 mRNA after BubR1 siRNA transfection; □, BubR1 siRNA (−); ■, BubR1 siRNA (+); data are mean ± S.D. (**C**) BubR1 siRNA reduced DSC1 expression and disarrayed HaCaT cells. Control siRNA-transfected HaCaT cells (panels a1, a2, a3, and a4) and BubR1 siRNA-transfected HaCaT cells (panels b1, b2, b3, and b4) were stained using BubR1- (green) and DSC1-specific antibodies (red) and analyzed by confocal laser scanning microscopy. DNA was visualized by DAPI staining (blue). Representative images are shown.

To investigate a possible mechanism of liver injury and impaired liver regeneration, we studied the effect of low BubR1 expression on desmocollin-1 (DSC1) expression in mouse liver tissue and HaCaT cells. Immunohistochemical examination revealed that DSC1 expression in the liver was attenuated after PHx in *BubR1*^*L/L*^ mice compared with *BubR1*^+/+^ mice (Fig. 4A). Western blot analysis of the liver extracts also revealed that in *BubR1*^*L/L*^ mice, DSC1 protein expression (0.36 ± 0.18, *P* < 0.05) was significantly decreased 24 h after PHx compared with *BubR1*^+/+^ mice (0.78 ± 0.26) (Fig. 4B). We then examined the effect of BubR1 siRNA on DSC1 expression in HaCaT cells. BubR1 expression was significantly decreased 24 and 48 h after transfection (siRNA(+) vs. siRNA(−) at 24 h; 0.21 ± 0.03 vs. 1.00 ± 0.19, *P* < 0.01, and at 48 h; 0.16 ± 0.05 vs. 0.89 ± 0.25, *P* < 0.01; Fig. 5A). DSC1 mRNA expression was also attenuated and significantly inhibited 48 h after transfection (siRNA(+) vs. siRNA(−); 1.19 ± 0.39 vs. 3.16 ± 0.89, *P* < 0.01; Fig. 5B). These results suggest that BubR1 regulates DSC1 expression in hepatocytes. To demonstrate the effect of BubR1 on junction formation in HaCaT cells treated with BubR1 siRNA and control siRNA, we performed confocal laser scanning microscopy analysis using combined BubR1/DSC1/DNA staining. Figure 5C shows confocal laser microphotographs of HaCaT cells treated with control siRNA (panels a1, a2, a3, and a4) and BubR1 siRNA (panels b1, b2, b3, and b4) 48 h after siRNA treatment. Panels a2 and a3 show a positive correlation between the expressions of BubR1 and DSC1. White arrows indicate high expressions of BubR1 and DSC1 in parallel (panels a2, a3, and a4). Yellow arrows indicate low expressions of BubR1 and DSC1 in parallel (panels a2, a3, a4, b2, b3, and b4). Compared with control siRNA-transfected cells (panel a3), BubR1 siRNA-transfected cells (panel b3) had a smaller number of red dot structures, which indicate desmosome junctions between cells (orange arrows). These confocal laser scanning microscopy findings suggest that BubR1 expression could be correlated with DSC1 expression and could therefore affect desmosome junction formation.

## Discussion

Aging delays liver regeneration after hepatectomy leading to postoperative liver failure and worsening general condition, which critically affect the patient’s prognosis. Although this phenomenon was reported over 50 years ago, the molecular mechanism for the loss of regenerative capacity in aged livers has not been fully elucidated^22^.

Our novel findings in this study are summarized as follows: (1) BubR1 insufficiency delays liver regeneration after PHx possibly because of transient impairment of G1-S cell cycle progression; (2) microscopic observation of *BubR1*^*L/L*^ mice liver shows increased necrotic hepatocytes. Transmission electron microphotographs of hepatic tissue also show increased necrotic hepatocytes accompanied by intercalated disc (ID) abnormality; and (3) we demonstrated, for the first time, that BubR1 controls expression of DSC1, a transmembrane cell adhesion protein in desmosomes. The increase in levels of DSC1, which is expressed in the desmosome, was suppressed in *BubR1*^*L/L*^ mice liver after PHx. In addition, down-regulation of BubR1 expression with siRNA reduced DSC1 expression in HaCaT cells. These findings provide evidence for a possible molecular mechanism by which aging impairs liver regeneration after hepatectomy.

BubR1 is a key molecule in the spindle assembly checkpoint during mitosis^1^. Previous studies demonstrated that BubR1 insufficiency in mice caused phenotypic changes that are similar to those found in aging mice^3^,^4^,^5^,^6^. At the early stage (48 h after PHx) of the liver regeneration, BubR1 mRNA expression was significantly increased in WT mice (*BubR1*^+/+^), suggesting that BubR1 is an important molecule involved in this process. Regeneration assessed by the increased LW/BW after PHx indicates impaired liver regeneration in *BubR1*^*L/L*^ mice. Aged WT mice also showed impaired liver regeneration, and we observed that BubR1 expression in intact liver significantly decreased in aged mice compared with young mice. These observations indicate that transient delayed liver regeneration after hepatectomy is associated with reduced BubR1 expression. Other factors, such as changes in EGF and FoxM1B expression, may also affect sustained delayed liver regeneration in aged mice^19^. It was previously observed in the senescent liver that activity of ALT, AST, and all types of bilirubin was increased in the blood of partially hepatectomized aged mice as a result of liver dysfunction^23^. The reduced BubR1 expression level itself did not affect circulating hepatic enzyme levels or liver weight and liver morphology in untreated *BubR1*^*L/L*^ mice (data not shown), ruling out the possibility that the reduction of BubR1 expression contributes to alterations of liver function and liver size in *BubR1*^*L/L*^ mice (Fig. 1D, Table 1). Thus, our results suggest that age-related inhibition of liver regeneration may be attributed to the reduction in BubR1 expression^17^,^24^,^25^.

Hepatocytes are normally quiescent cells. The initiation of hepatocyte proliferation or liver regeneration after hepatectomy requires activation of the mitogenic genes and repression of genes responsible for inhibiting hepatocyte proliferation^26^. Fewer hepatocytes in older animals and humans enter the S-phase after partial hepatectomy compared with younger subjects^19^. In mice, a sharp peak of hepatocyte proliferation is usually observed at 48 h post-PHx^20^, but such steep increase and decrease were not observed in *BubR1*^*L/L*^ mice. Instead, a few cells initiated proliferation and mitosis 48 h after PHx, and continued to proliferate up to 144 h. These findings suggest that hepatocyte proliferation after PHx was delayed in *BubR1*^*L/L*^ mice. As a result, delayed cyclin gene expression was observed in hepatectomized *BubR1*^*L/L*^ mice. Comparison of cyclin-D expression patterns in *BubR1*^+/+^ and *BubR1*^*L/L*^ mice revealed an apparent delay in cyclin-D induction after PHx in *BubR1*^*L/L*^ mice. Increased cyclin-D and -E expression levels accelerate the G1/S phase transition^27^. At 24 h after PHx, cyclin-D and -E expression levels in *BubR1*^*L/L*^ mice were higher than those in *BubR1*^+/+^ mice, suggesting that G1/S phase cells may accumulate in *BubR1*^L/L^ mice. Cyclin-A and -B expression levels increase in late S phase and peak in G2 of normal cells^28^. While cyclin-A and -B peak expression levels were observed at 48 h after PHx in *BubR1*^+/+^ mice, moderate cyclin-A and -B expression was induced at 48 h after PHx in *BubR1*^*L/L*^ mice, and remained constant up to 144 h. These patterns are the same as those of the mitotic and PCNA-positive indices hepatocytes in hepatectomized *BubR1*^*L/L*^ mice. These results suggested that fewer proliferating hepatocytes may result from a delay in cell cycle progression in *BubR1*^*L/L*^ mouse hepatocytes. We also found that p21 expression was higher in *BubR1*^*L/L*^ than *BubR1*^+/+^ mice at 24 h post-PHx. p21 is a CDK inhibitor that hampers the activity of cyclin-E/CDK2 and cyclin-D/CDK4/6 complexes^29^. Therefore, our results suggest that arrest and/or delay of cell cycle progression from G1 to S phase occurred in hepatocytes in the partially hepatectomized *BubR1*^*L/L*^ mice. Several studies demonstrate that p21 plays a key role during liver regeneration, such as in the acute liver injury model^30^ and in the extended hepatectomy model^20^. Moreover, there was no difference in the HGF protein levels in *BubR1*^+/+^ mice and *BubR1*^*L/L*^ mice. This fact suggests that both types of mice received equivalent levels of stimulation of cell proliferation. Therefore these studies demonstrated up-regulation of p21 accompanied by a delay in cell cycle progression, which is consistent with our results in *BubR1*^*L/L*^ mice.

Microscopic observation revealed that after PHx, necrosis but not apoptosis of hepatocytes was strongly induced in *BubR1*^*L/L*^ mice compared with *BubR1*^+/+^ mice. These observations were consistent with biochemical analysis of blood in *BubR1*^*L/L*^ mice. The activities of ALT, AST and all types of bilirubin were increased in partially hepatectomized *BubR1*^*L/L*^ mice. Such increases are observed in the clinical condition when liver regeneration is impaired in the patients after PHx. In the regenerating liver, epithelial cells require physical contact with extracellular matrix to avoid the detachment induced cell death (DICD) of hepatocytes. After PHx, the reconstitution of a perisinusoidal extracellular matrix stabilizes the newly formed hepatocyte population. Thus, necrosis that is observed in partially hepatectomized *BubR1*^*L/L*^ mice might be the result of excessive DICD caused by failure of perisinusoidal extracellular matrix reconstitution.

Morphological analysis of livers from *BubR1*^*L/L*^ mice demonstrated a widened interhepatocyte space, shrinking hepatocytes, a widened perisinusoidal space and atrophy of microvilli in the early stages after PHx, suggesting that partial microstructure failure results from a weak adaptation to changed microcirculatory flow by PHx in *BubR1*^*L/L*^ mice. Hepatocytes connect via intercellular junctions called IDs to form liver cell plates. IDs are composed of different types of junctional complexes, including gap-junctions made of connexins, tight- and adherens-junctions, and desmosomes. Desmosomes are button-like structures composed of intracellular anchor proteins and transmembrane adhesion proteins. The intracellular anchor proteins play a role in connecting the cytoskeleton to the transmembrane adhesion proteins including desmocollin, a member of the cadherin family. Desmosomes maintain the integrity of cells to resist mechanical stress, and have dynamic structures whose adhesiveness can switch between high and low affinity adhesive states during cell division, thereby participating in fundamental processes such as cell proliferation, differentiation and morphogenesis^31^. Thus, it is possible that the weakened cell attachment observed in the hepatectomized *BubR1*^*L/L*^ mice could be caused by desmosome abnormality. Hepatocyte junctions are made up of multiple components. We evaluated the expressions of occludin (located in tight junctions), E-cadherin (located in adherent junctions), DSC1, and desmoglein in mouse liver. Immunohistochemical analysis revealed that the expressions of occludin, E-cadherin, and desmoglein were not markedly different between *BubR1*^*L/L*^ mice and *BubR1*^+/+^ mice (data not shown). However, the expression of DSC1 was markedly different between control cells and BubR1-reduced cells *in vitro* and in mouse liver. DSC1, a desmosome transmembrane cell adhesion protein, is highly expressed in the liver^32^. We showed that up-regulation of DSC1 expression after PHx was not observed in *BubR1*^*L/L*^ mice. We also demonstrated that siRNA-mediated down-regulation of BubR1 suppressed DSC1 expression in HaCaT cells, in which it is well known that both DSC1 mRNA and protein are abundant. These results suggest that BubR1 could be a critical regulating factor for DSC1 expression. We suggest that DSC1 could be a target protein which is ubiquitinated by APC/C thereby marking it for degradation by the proteasome. To investigate this hypothesis, we examined DSC1 protein expression under an APC/C inhibitor using western blot analysis. This analysis showed that the APC/C inhibitor upregulated DSC1 expression in the same way as the inhibitor upregulates securin, with which BubR1 directly interacts *in vitro* and *in vivo* (data not shown). Consequently, DSC1 may be one of the proteins ubiquitinated by APC/C and, therefore, may be a target of BubR1. However, further research on the relationship between cell cycle checkpoint proteins and junction proteins is needed. The reduced DSC1 level could cause abnormal cell attachment and delay liver regeneration in *BubR1*^*L/L*^ mice.

Based on our findings, we propose a possible scenario that occurs after PHx in aged individuals. Hepatectomy induces many stresses, including physical stress and cell division-associated stress. After hepatic resection, portal hypertension occurs because the vascular network in the remnant lobes is reduced despite constant blood flow from the portal vein^33^. The liver cells and liver sinusoidal endothelial cell attachment are affected by these stresses during liver regeneration after hepatectomy^34^, leading to transient DICD and G1 arrest to prevent an excessive DICDs. DSC1 expression also increases, thus leading to reinforcement of desmosomes to maintain cell attachments, which is an important process to prevent massive DICD and anokisis, and thus focal necrosis of hepatocytes. In the case of low BubR1 expression, the increased level of DSC1 expression is not enough to reinforce the desmosome after PHx, leading to increased DICD and anokisis, and thus resulting in hepatocyte focal necrosis. Impairment of cell attachment induces DICD and G1 arrest in a p21-dependent manner^35^. Therefore, the delayed G1/S progression occurs through up-regulation of p21 caused by secondary effects from the failure of DICD reinforcement because of a DSC1 abnormality, rather than via a direct effect of low BubR1 levels. Thus, delayed liver regeneration could occur after PHx in aged patients.

In summary, BubR1 insufficiency causes delayed liver regeneration after partial hepatectomy. BubR1 expression is reduced along with aging. BubR1 insufficiency reduces DSC1 expression in the liver, and thus weakens the microstructural adaptation of the tissue against hepatectomy. BubR1 insufficiency, thus, enhances transient liver failure and injury through the cell cycle delay caused by up-regulation of p21 that results from the weakened microstructural adaptation. Therefore, hepatocyte proliferation is delayed and liver regeneration could be impaired. This work provides new insights into the role of BubR1 in liver regeneration, which may explain worsening liver function after hepatectomy in aged patients.

## Methods

Animals were maintained under standard conditions and treated according to the Guidelines for the Care and Use of Laboratory Animals of Kyushu University. All experimental protocols were approved by the Kyushu University Institutional Animal Care and Use Committee. (Approval no. A24-220-0).

### Experimental animals

Low-*BubR1*-expressing mutant (*BubR1*^*L/L*^) and wild-type (WT) littermate control (*BubR1*^+/+^) mice on a mixed genetic background of C57BL/6J/ and 129/SvJ were generated in our laboratory, as previously described^7^. Five-week-old *BubR1*^*L/L*^ and *BubR1*^+/+^ male mice were used for experiments. Young (7-week-old) and aged (53-week-old) C57BL/6JJcl male mice were obtained from CLEA Japan Inc. (Tokyo, Japan), and kept in our facility for 2 weeks before use.

### Partial hepatectomy model

Seventy percent PHx was performed as previously described^17^,^36^. Briefly, the bilateral median lobe and left lateral lobe were ligated and removed. After the wounds were sutured, animals were kept on a warming mat to avoid hypothermia. Mice were sacrificed to collect the remaining lobes and blood samples at each indicated time point before and after PHx. The liver weight-to-body weight ratio (LW/BW) was calculated to estimate liver mass recovery. Liver tissue samples were frozen or fixed with formalin and embedded in paraffin. Blood samples were collected from the postcava during anesthesia.

### Biochemical analyses

Asparate:2-oxoglutarate aminotransferase (AST), alanine:2-oxoglutarate aminotransferase (ALT), lactate dehydrogenase (LDH), total bilirubin (T-BIL), direct bilirubin (D-BIL), indirect bilirubin (I-BIL), total bile acid (TBA), and alkaline phosphatase (ALP) plasma levels were analyzed by Nagashima Life Sciences Laboratory (Shiga, Japan).

### Cell culture

A human keratinocyte cell line (HaCaT, Cell Lines Service, Eppelheim, Germany) was cultured in Dulbecco’s Modified Eagle’s Medium (DMEM, Thermo Fisher Scientific Inc., Waltham, MA, USA), supplemented with 10% heat-inactivated fetal bovine serum (FBS, Sigma-Aldrich Corp, St. Louis, MO, USA), 100 U/ml penicillin and 100 μg/ml streptomycin (Thermo Fisher Scientific Inc.) in plastic disposable tissue culture flasks for RNA extraction and on coverslips for confocal laser scanning microscopy at 37 °C in a 5% CO_2_/95% air incubator.

### BubR1 knockdown using siRNA

Lipofectamine RNAiMAX transfection reagent (Life Technologies, Carlsbad, CA, USA) was used to transfect a siRNA targeting BubR1 (sc-37542; Santa Cruz Biotechnology, Dallas, TX, USA) or negative control (12935-112; Thermo Fisher Scientific Inc.) into HaCaT cells at a final concentration of 5 nmol/L in transfection reagent (dilution, 1:1000; vol/vol), according to the manufacturer’s protocol. After siRNA transfection at 24, 48 and 72 h, siRNA-transfected HaCaT cells were collected. Total RNA was extracted and reverse-transcription was performed to obtain cDNA. BubR1 and desmocollin-1 (DSC1) mRNA expression was evaluated using quantitative real-time polymerase chain reaction (qRT-PCR). For confocal laser scanning microscopy, cells were fixed with ice-cold methanol 48 h after siRNA transfection.

### mRNA quantification using qRT-PCR

Total RNA was isolated using ISOGEN (Nippongene, Tokyo, Japan) and cDNAs were synthesized from RNAs using SuperScript III First-Strand Synthesis SuperMix (Thermo Fisher Scientific Inc.) according to the manufacturer’s instructions. qRT-PCR amplification was performed using Applied Biosystems StepOnePlus Real-Time PCR System (Thermo Fisher Scientific Inc.). The primer/probe sets for mouse Ccnd1 (Mm00432359_m1), Ccne1 (Mm00432367_m1), Ccna2 (Mm00438064_m1), Ccnb2 (Mm01171453_m1), p21 (Mm04205640_g1), p16 (Mm00494449_m1) and desmocollin-1 (Mm00496525_m1) from the TaqMan gene expression assays (Thermo Fisher Scientific Inc.) and the primer/probe set for β-actin (Mm00607939_s1) as a mouse endogenous control were purchased from Thermo Fisher Scientific Inc. The primer/probes sets for human desmocollin-1 (Hs00245189_m1) and BubR1 (Hs02758991_g1) from the TaqMan gene expression assays (Thermo Fisher Scientific Inc.) and the primer/probe set for glyceraldehyde-3-phosphate dehydrogenase (GAPDH, Hs02758991_g1) as a human endogenous control were purchased from Applied Biosystems^17^. Mouse BubR1 expression was analyzed using QuantiFast SYBR Green PCR kit (QIAGEN, Tokyo, Japan) and normalized to GAPDH, as previously described^7^. The sequences of primers are as follows: mouse BubR1, 5′-CAG TCC CAG CAC AGA CAG TTC CA-3′ (forward) and 5′-GCT AGC GAG CTT CTC TGT GGT TCA-3′ (reverse); and GAPDH 5′-ATC TGG AAA GCT GTG GCG-3′ (forward) and 5′-CCA CGA CGG ACA CAT TG-3′ (reverse).

### Tissue histopathological examination

Hematoxylin-eosin (HE) staining and silver impregnation stain were performed on 10% formalin-fixed and 3-μm paraffin-embedded sections of liver tissue. For transmission electron microscopic observation, liver samples were fixed in glutaraldehyde. Immunostaining was performed to detect proliferating cell nuclear antigen (PCNA; M0879, Dako, Tokyo, Japan) and streptavidin–biotin–peroxidase staining was performed using Histofine SAB-PO (M) immunohistochemical staining kit (Nichirei Bioscience Inc., Tokyo, Japan) and counterstained with hematoxylin. At least 1000 hepatocytes were counted for the mitotic index and PCNA positivity in different sections for each group. The necrosis score was calculated by counting the number of necrotic lesions per low-power magnification (×40) in 8 fields per slide.

Formalin-fixed/paraffin-embedded tissue specimens were used for immunofluorescence staining. Deparaffinization was performed by soaking in xylene for 5 min (twice), 100% ethanol for 3 min, 95% ethanol for 3 min, 85% ethanol for 3 min, 75% ethanol for 3 min and washing with PBS for 5 min. The sections were pretreated by autoclaving (121 °C) for 20 min in 0.01 mol/L citrate-buffered saline (pH 6.0) for antigen retrieval. Non-specific reactions were blocked with 10% goat serum (Histofine SAB-PO(R) kit, Nichirei Bioscience Inc.) for 10 min. The sections were incubated with rabbit polyclonal antibodies against DSC1 (LS-C167539, 1:50; LifeSpan BioSciences, Seattle, WA, USA) at 4 °C overnight, before washing with PBS. The sections were incubated with anti-rabbit immunofluorescence antibodies (Alexa Fluor 555, 1:200, Abcam) to detect DSC1 for 60 min and washed 3 times with PBS for 5 min each.

### Confocal laser scanning microscopy

siRNA-transfected HaCaT cells were rinsed in PBS at 37 °C, fixed in cold methanol for 3 min at −20 °C, blocked for 30 min at room temperature using PBS containing 2% BSA and 2% normal goat serum, and incubated with the following antibodies at the indicated dilutions: rat anti-DSC1 at 1:100 (MAB7367; R&D Systems, Inc., Minneapolis, MN, USA), rabbit anti-BubR1 at 1:100 (612503; BD Biosciences, San Jose, CA, USA). Cells were rinsed in PBS at 37 °C, fixed in 4% paraformaldehyde for 15 min at 37 °C, permeabilized for 5 min at room temperature using PBS containing 0.2% Triton X-100, and blocked for 30 min at room temperature using PBS containing 2% BSA and 2% normal goat serum. Secondary antibodies conjugated to Alexa Fluor 488 and 555 (Molecular Probes) were used at 1:2,000 dilution. After being washed in PBS containing 4,6-diamidino-2-phenylindole (DAPI) for 5 min, the coverslips were mounted in ProLong Gold (Thermo Fisher Scientific Inc). Fluorescence image acquisition was performed using a Nikon A1R confocal imaging system controlled by Nikon NIS Elements software (Nikon). The objective lens was an oil immersion Plan-Apo ×60 numerical aperture 1.40 lens (Nikon). Images were acquired as Z-stacks at 0.2-μm intervals, and maximum-intensity projections were generated using NIS Elements.

### Western blot analysis

Western blot analysis was performed as previously described^37^. Livers were isolated and all surrounding material was removed. After preparing lysates, proteins were separated using SDS-PAGE and transferred to a nylon membrane. The blots were probed with primary antibody for DSC1 (LS-C 167539, 1:500, LifeSpan BioSciences). Equal loading was confirmed using β-actin (#4970, 1:1000, Cell Signaling Technology Inc.). Results are representative of three independent animals of each genotype at each age.

### Enzyme-linked immunosorbent assay for hepatocyte growth factor

Frozen liver tissue was homogenized in lysis buffer (CelLytic MT Cell Lysis Reagent, Sigma Aldrich, St. Louis, MO, USA) containing protease inhibitor cocktail (Nacalai Tesque, Kyoto, Japan) and hepatic hepatocyte growth factor (HGF) levels were measured using the Mouse/Rat HGF Quantikine ELISA Kit (R&D Systems, Minneapolis, MN, US). HGF levels were normalized to total protein levels in the liver tissue, as measured using a Bio-Rad Protein Assay (Bio-Rad, Hercules, CA, USA).

### Statistical analysis

Data are presented as the mean ± standard deviation (SD). The Student’s *t*-test was performed using JMP pro (version 9.0.0, SAS Institute Inc., Cary, NC, USA). Additional professional statistical assistance was provided by Junji Kishimoto, Kyushu University. Differences were considered to be significant at *P* < 0.05.

## Additional Information

**How to cite this article**: Ikawa-Yoshida, A. *et al*. BubR1 Insufficiency Impairs Liver Regeneration in Aged Mice after Hepatectomy through Intercalated Disc Abnormality. *Sci. Rep.* **6**, 32399; doi: 10.1038/srep32399 (2016).

## Figures and Tables

**Table 1:** Serial change in laboratory data of BubR1^+/+^ and BubR1^L/L^ mice.

*BubR1*^+/+^							*BubR1*^*L/L*^					
	Intact	After 70% PHx (hrs)					Intact	After 70% PHx (hrs)				
		12	24	48	96	144		12	24	48	96	144
AST(IU/L)	78 ± 28	5202 ± 1539^††^	1777 ± 1307^†^	174 ± 72^†^	82 ± 16	62 ± 9	43 ± 5	7733 ± 1811^††^*	7006 ± 3134^††^*	1174 ± 1210	79 ± 17^††^	61 ± 10^†^
ALT(IU/L)	42 ± 21	4110 ± 1313^††^	2044 ± 1188^††^	179 ± 117	29 ± 6	23 ± 4	22 ± 4	5853 ± 1025^††^*	4634 ± 2015^††^*	535 ± 522	29 ± 6	22 ± 3
LDH(IU/L)	359 ± 134	11159 ± 3563^††^	3497 ± 26734^†^	376 ± 156	279 ± 40	252 ± 32	248 ± 70	15555 ± 2560^††^*	14360 ± 8265^†^*	1880 ± 2015	402 ± 150	307 ± 39*
T-BIL(mg/dL)	0.06 ± 0.01	0.15 ± 0.02^††^	0.19 ± 0.23	0.12 ± 0.07	0.13 ± 0.07	0.06 ± 0.02	0.10 ± 0.03	0.87 ± 0.92	4.22 ± 2.82^†^*	5.28 ± 6.97	0.15 ± 0.03^†^	0.11 ± 0.04*
D-BIL(mg/dL)	0.04 ± 0.02	0.04 ± 0.03	0.10 ± 0.18	0.05 ± 0.04	0.08 ± 0.05	0.02 ± 0.01	0.05 ± 0.01	0.62 ± 0.79	3.26 ± 2.17^†^*	4.17 ± 5.61	0.05 ± 0.02	0.05 ± 0.01**
I-BIL(mg/dL)	0.02 ± 0.02	0.11 ± 0.02^††^	0.09 ± 0.05^†^	0.07 ± 0.04	0.05 ± 0.03	0.05 ± 0.01^†^	0.06 ± 0.04	0.25 ± 0.13^†^*	0.96 ± 0.67^†^*	1.11 ± 1.36	0.10 ± 0.02*	0.06 ± 0.03
TBA(μmol/L)	16.8 ± 31.5	11.6 ± 6.2	24.1 ± 38.1	10.4 ± 6.7	4.7 ± 4.5	2.2 ± 1.0	24.8 ± 47.5	383.8 ± 563.3	1216.2 ± 728.8^†^*	819.0 ± 905.1	5.2 ± 1.1	3.4 ± 1.3
ALP(IU/L)	469 ± 54	843 ± 188^††^	921 ± 324^††^	461 ± 49	308 ± 58^††^	413 ± 96	482 ± 52	851 ± 437	2725 ± 1231^†^*	3270 ± 3832	273 ± 96^††^	328 ± 88^†^

PHx, partial hepatectomy; AST, asparate: 2-oxoglutarate aminotransferase; ALT, alanine: 2-oxoglutarate aminotransferase; LDH, lactate dehydrogenase; T-BIL, total bilirubin; D-BIL, direct bilirubin; I-BIL, indirect bilirubin; TBA, total bile acid; ALP, alkaline phosphatase. ^†^p < 0.05, ^††^p < 0.01 vs each intact, *p < 0.05, **p < 0.01 vs *BubR1*^+/+^ mice.

**Table 2:** Serial change in laboratory data of young and old mice.

	young (9-week-old)					aged (55-week-old)				
	intact	After 70% PHx (hrs)				intact	After 70% PHx (hrs)			
		24	48	96	144		24	48	96	144
AST(IU/L)	30 ± 2	2640 ± 1029^††^	191 ± 50^††^	94 ± 16^††^	77 ± 31	52 ± 28**	5126 ± 1212^††^*	402 ± 67^††^**	87 ± 8	71 ± 6
ALT(IU/L)	15 ± 2	2528 ± 728^††^	169 ± 56^†^	36 ± 7^††^	34 ± 15	31 ± 19**	4451 ± 999^††^*	611 ± 132^††^**	51 ± 12	28 ± 5
LDH(IU/L)	185 ± 18	6299 ± 3072^†^	382 ± 70^††^	527 ± 102^††^	228 ± 92	305 ± 61*	13592 ± 5059^†^*	645 ± 60^††^**	314 ± 5*	325 ± 121

PHx, partial hepatectomy; AST, asparate: 2-oxoglutarate aminotransferase; ALT, alanine: 2-oxoglutarate aminotransferase; LDH, lactate dehydrogenase. ^†^p < 0.05, ^††^p < 0.01 vs each intact, *p < 0.05, **p < 0.01 vs young mice.

## References

[b1] Yu H. Regulation of APC-Cdc20 by the spindle checkpoint. Current opinion in cell biology. 14, 706–714 (2002). 12473343 10.1016/s0955-0674(02)00382-4

[b2] Bolanos-Garcia V. M. & Blundell T. L. BUB1 and BUBR1: multifaceted kinases of the cell cycle. Trends Biochem Sci. 36, 141–150 (2011). 20888775 10.1016/j.tibs.2010.08.004 PMC3061984

[b3] Baker D. J. . BubR1 insufficiency causes early onset of aging-associated phenotypes and infertility in mice. Nature genetics 36, 744–749 (2004). 15208629 10.1038/ng1382

[b4] Baker D. J. . Opposing roles for p16Ink4a and p19Arf in senescence and ageing caused by BubR1 insufficiency. Nature cell biology 10, 825–836 (2008). 18516091 10.1038/ncb1744 PMC2594014

[b5] Baker D. J. . Clearance of p16Ink4a-positive senescent cells delays ageing-associated disorders. Nature 479, 232–236 (2011). 22048312 10.1038/nature10600 PMC3468323

[b6] Matsumoto T. . Aging-associated vascular phenotype in mutant mice with low levels of BubR1. Stroke 38, 1050–1056 (2007). 17272762 10.1161/01.STR.0000257967.86132.01

[b7] Kyuragi R. . BubR1 insufficiency inhibits neointimal hyperplasia through impaired vascular smooth muscle cell proliferation in mice. Arterioscler Thromb Vasc Biol. 35, 341–347 (2015). 25524773 10.1161/ATVBAHA.114.304737

[b8] Michalopoulos G. K. & DeFrances M. C. Liver regeneration. Science 276, 60–66 (1997). 9082986 10.1126/science.276.5309.60

[b9] Fausto N. & Webber E. M. In The Liver Biology and Pathobiology (eds Arias I. M., Boyer J. L., Fausto N., Jacoby W. B., Schachter D., Shafritz D.A. ) (Raven Press Ltd, New York, USA) 53–68 (1994).

[b10] Taub R. Liver regeneration: from myth to mechanism. Nat Rev Mol Cell Biol. 5, 836–847 (2004). 15459664 10.1038/nrm1489

[b11] Matsuo T. . Control mechanism of the circadian clock for timing of cell division *in vivo*. Science 302, 255–259 (2003). 12934012 10.1126/science.1086271

[b12] Rudolph K. L. . Inhibition of experimental liver cirrhosis in mice by telomerase gene delivery. Science 287, 1253–1258 (2000). 10678830 10.1126/science.287.5456.1253

[b13] Satyanarayana A. . Telomere shortening impairs organ regeneration by inhibiting cell cycle re-entry of a subpopulation of cells. EMBO J 22, 4003–4013 (2003). 12881434 10.1093/emboj/cdg367 PMC169040

[b14] Grisham J. W. A morphologic study of deoxyrobonucleic acid synthesis and cell proliferation in regenerating rat liver:autoradiography with thymidine-H. Cancer Res. 22, 842–849 (1962). 13902009

[b15] Guicciardi M. E., Malhi H., Mott J. L. & Gores G.J. Apoptosis and necrosis in the liver. Compr Physiol. 3, 977–1010 (2013). 23720337 10.1002/cphy.c120020 PMC3867948

[b16] Fausto N., Campbell J. S. & Riehle K. J. Liver regeneration. Hepatology 43, 45–53 (2006). 10.1002/hep.20969 16447274

[b17] Haga S. . p66(Shc) has a pivotal function in impaired liver regeneration in aged mice by a redox-dependent mechanism. Laboratory investigation; a journal of technical methods and pathology 90, 1718–1726 (2010). 10.1038/labinvest.2010.119 20567235

[b18] Fortner J. G. & Lincer R. M. Hepatic resection in the elderly. Ann. Surg. 211, 141–145 (1990). 2301993 10.1097/00000658-199002000-00005 PMC1357957

[b19] Schmucker D. L. & Sanchez H. Liver Regeneration and Aging. A Current Perspective.Curr Gerontol Geriatr Res. 2011, 526379 (2011). 21912543 10.1155/2011/526379 PMC3170699

[b20] Lehmann K. . Liver Failure After Extended Hepatectomy in Mice Is Mediated by a p21-Dependent Barrier to Liver Regeneration. Gastroenterology 143, 1609–1619 e1604 (2012). 22960658 10.1053/j.gastro.2012.08.043

[b21] Michalopoulos G. K. Liver regeneration. J Cell Physiol. 213, 286–300 (2007). 17559071 10.1002/jcp.21172 PMC2701258

[b22] Timchenko N. A. Aging and liver regeneration. Trends Endocrinol Metab. 20, 171–176 (2009). 19359195 10.1016/j.tem.2009.01.005

[b23] Schmucker D. L. Age-related changes in liver structure and function: Implications for disease? Exp Gerontol. 40(**8–9**), 650–659 (2005). 16102930 10.1016/j.exger.2005.06.009

[b24] Gregg S. Q. . A mouse model of accelerated liver aging caused by a defect in DNA repair. Hepatology. 155, 609–621 (2012). 21953681 10.1002/hep.24713 PMC3250572

[b25] Sánchez-Hidalgo J. M. . Impact of age on liver regeneration response to injury after partial hepatectomy in a rat model. J Surg Res. 175(1), e1–e9 (2012). 22341343 10.1016/j.jss.2011.11.1022

[b26] Su A. I. . Gene expression during the priming phase of liver regeneration after partial hepatectomy in mice. Proc Natl Acad Sci USA 99, 11181–11186 (2002). 12177410 10.1073/pnas.122359899 PMC123230

[b27] Resnitzky D., Gossen M., Bujard H. & Reed S. I. Acceleration of the G1/S phase transition by expression of cyclins D1 and E with an inducible system. Mol Cell Biol. 14(3), 1669–1679 (1994). 8114703 10.1128/mcb.14.3.1669-1679.1994 PMC358525

[b28] Muschel R. J., Zhang H. B. & McKenna W. G. Differential Effect of Ionizing Radiation on the Expression of Cyclin A and Cyclin B in Hela Cells. Cancer Res. 53, 1128–1135 (1993). 8439958

[b29] Wang X., Kiyokawa H., Dennewitz M. B. & Costa R. H. The Forkhead Box m1b transcription factor is essential for hepatocyte DNA replication and mitosis during mouse liver regeneration. Proceedings of the National Academy of Sciences of the United States of America 99, 16881–16886 (2002). 12482952 10.1073/pnas.252570299 PMC139238

[b30] Hui T. T. . Immediate early genes and p21 regulation in liver of rats with acute hepatic failure. Am J Surg. 183, 457–463 (2002) 11975936 10.1016/s0002-9610(02)00822-x

[b31] Garrod D. & Chidgey M. Desmosome structure, composition and function. Biochim Biophys Acta 1778, 572–587 (2008). 17854763 10.1016/j.bbamem.2007.07.014

[b32] Donetti E. . Desmocollin 1 expression and desmosomal remodeling during terminal differentiation of human anagen hair follicle: an electron microscopic study.Exp Dermatol. 13(5), 289–297 (2004). 15140019 10.1111/j.0906-6705.2004.00152.x

[b33] Bosch J. . Pathophysiology of portal hypertension. Gastroenterol Clin North Am. 21, 1–14 (1992). 1568769

[b34] Sato Y., Koyama S., Tsukada K. & Hatakeyama K. Acute portal hypertension reflecting shear stress as a trigger of liver regeneration following partial hepatectomy. Surg Today. 27, 518–526 (1997). 9306545 10.1007/BF02385805

[b35] Collins N. L. . G1/S cell cycle arrest provides anoikis resistance through Erk-mediated Bim suppression. Mol Cell Biol. 25, 5282–5291 (2005). 15923641 10.1128/MCB.25.12.5282-5291.2005 PMC1140593

[b36] Greene A. K. & Puder M. Partial hepatectomy in the mouse: technique and perioperative management. Journal of investigative surgery. The official journal of the Academy of Surgical Research 16, 99–102 (2003). 12746193

[b37] Liu X. . Leonurine protects against tumor necrosis factor-alpha-mediated inflammation in human umbilical vein endothelial cells. Atherosclerosis 222, 34–42 (2012). 22326051 10.1016/j.atherosclerosis.2011.04.027

